# Beyond CE Marking: The Need for Life-Cycle Health Technology Assessment of Medical Devices for Patient Safety and Health-System Value

**DOI:** 10.3390/healthcare14142179

**Published:** 2026-07-19

**Authors:** Christos Ntais, Michael A. Talias

**Affiliations:** Healthcare Management Program, School of Economics & Management, Open University of Cyprus, Nicosia 2220, Cyprus; michael.talias@ouc.ac.cy

**Keywords:** health technology assessment, medical devices, CE marking, patient safety, cost-effectiveness, real-world evidence, hospital-based HTA, procurement, post-market surveillance, life-cycle HTA

## Abstract

Background/Objectives: Medical devices are essential to modern healthcare, but their adoption is often driven by regulatory conformity, clinical enthusiasm, procurement pressures and vendor-led innovation rather than systematic evaluation of comparative value. CE marking and related regulatory mechanisms are necessary for market access; however, they do not determine whether a device improves patient-related outcomes compared with existing alternatives, whether its benefits justify its total costs, or whether it can be implemented safely in routine care. This narrative review examines why medical devices require a dedicated life-cycle health technology assessment (HTA) approach and proposes an operational framework linking assessment to adoption, evidence generation, reassessment and disinvestment. Methods: A structured targeted search covered peer-reviewed literature and policy or institutional documents addressing HTA, medical devices, regulation, economic evaluation, real-world evidence, hospital-based HTA, procurement digital and AI-enabled devices, patient involvement and post-market reassessment. Results: Medical devices differ from pharmaceuticals through user dependence, learning curves, procedure dependence, short product life cycles, incremental modification, heterogeneous comparators, limited randomized evidence and hidden life-cycle costs. These features create clinical, economic, organizational and implementation uncertainty after market entry. The proposed model specifies six linked phases: horizon scanning and early dialogue, pre-adoption appraisal, an explicit adoption decision, controlled implementation, real-world monitoring and scheduled or trigger-based reassessment leading to continuation, scale-up, restriction, or disinvestment. Practical constraints include fragmented data infrastructure, the cost of maintaining registries and residual confounding in real-world evidence. Conclusions: Medical device HTA should move beyond one-time pre-adoption assessment toward a decision-linked life-cycle model that integrates comparative value, patient and public involvement, procurement, implementation governance, real-world evidence, version monitoring, reassessment and disinvestment. This approach can support responsible innovation, patient safety, transparent procurement and sustainable health-system value.

## 1. Introduction

Medical devices play a vital role in contemporary healthcare. They support diagnosis, treatment, monitoring, rehabilitation, prevention and, increasingly, digital and AI-enabled care. The term covers a heterogeneous group of technologies, from relatively simple consumables to implantable devices, imaging systems, in vitro diagnostics, robotic platforms, remote monitoring tools, clinical decision-support software and Software as a Medical Device (SaMD). This diversity creates major challenges for decision-makers. For example, a hospital may need to decide whether to purchase a new surgical platform, a payer may need to decide whether to reimburse an implantable device, and a national health technology assessment agency may need to assess whether a diagnostic technology improves overall health outcomes along an entire care pathway.

Health Technology Assessment (HTA) represents a systematic method for helping decision makers make informed decisions when assessing health technologies. HTA is defined as a multidisciplinary process that uses explicit methods to determine the value of a health technology at different points in its life cycle, with the purpose of supporting equitable, efficient and high-quality health systems [1,2]. The World Health Organization (WHO) has emphasized that HTA can support decision-making on the adoption and reimbursement of medical technologies by evaluating their clinical, economic, ethical, social and organizational implications [3]. In principle, HTA is therefore not limited to pharmaceuticals. It could apply to medicines, vaccines, devices, diagnostics, procedures, programs and systems.

In practice, however, HTA is generally more mature and entrenched for pharmaceuticals than for medical devices. Pharmaceuticals usually follow relatively standardized pathways for regulatory approval, price negotiation, reimbursement and post-market evidence generation. Conversely, medical devices often enter clinical practice through multiple routes, including hospital procurement, departmental purchasing, leasing agreements, donations, capital investment cycles, physician preference, or vendor service contracts. Previous research suggests that differences in diffusion reflect the heterogeneity of devices, the local pathways through which they are adopted and the limited comparative evidence available at the point of diffusion [4,5,6,7]. Consequently, devices may diffuse locally before sufficient evidence is available on their comparative clinical benefit, economic value, implementation requirements and long-term performance. This is particularly common for hospital-based technologies, which may be in routine use before a national HTA organization has produced a comprehensive assessment.

This gap is important since regulatory approval and HTA answer different questions. Regulatory approval, including CE marking in the European Union, essentially determines whether a device meets safety and performance requirements for its intended purpose under the medical device regulatory framework [8]. HTA addresses the broader question of whether a device should be adopted into a particular healthcare system, reimbursed by third party payers, procured by organizations using public funds, scaled up, restricted, monitored further, or discontinued in a specific health system. This question requires evidence on the comparative effectiveness of a technology across patient populations, patient-related outcomes, safety data generated from studies conducted in routine clinical practice, cost-effectiveness analyses, estimated budget impact, evaluation of organizational readiness to implement the technology, evaluation of equity issues, and the assessment of ethical considerations and long-term value.

The European policy context makes this issue particularly timely. Regulation (EU) 2021/2282 established an overarching framework for joint clinical assessments at the EU level and took effect on 12 January 2025 [9]. Joint clinical assessments are intended to support national HTA processes by providing scientific analysis of clinical evidence on the relative effects of health technologies, including pharmaceuticals and medical devices [10]. Initially, the mandatory scope covers new oncology medicines and advanced therapy medicinal products. According to the 2026 Work Programme of the Member State Coordination Group on HTA, selected high-risk medical devices are expected to enter joint clinical assessment from 2026 onwards [11]. In October 2025, the European Commission also published Implementing Regulation (EU) 2025/2086 on the joint clinical assessment of medical devices and in vitro diagnostic medical devices [12]. This evolving framework reinforces the primary objective of this review: to explain why medical devices should no longer be viewed mainly through regulatory conformity but should undergo a dedicated life-cycle HTA process that evaluates their clinical, economic, organizational, patient and real-world value.

The individual challenges of medical device assessment—including learning curves, user dependence, rapid iteration, limited randomized evidence and hidden costs—are already well established. The original contribution of this review is therefore not to claim that these features are newly identified, but to integrate them into an operational decision-linked life-cycle HTA framework. The framework specifies phases, stakeholders, evidence inputs, reassessment triggers and possible decisions and connects national assessment with reimbursement, hospital governance, procurement, patient and public involvement, and disinvestment. It also extends life-cycle HTA to versioned and AI-enabled devices, for which the assessed technology may change after deployment.

## 2. Materials and Methods

This article was designed as a narrative review and policy-oriented synthesis. A structured targeted search was used to identify peer-reviewed articles, policy documents, regulations and institutional guidance relevant to the HTA of medical devices. Searches covered PubMed and Scopus and were supplemented by targeted searches of official websites from the WHO, European Commission, Medical Device Coordination Group, EU-level HTA initiatives, National Institute for Health and Care Excellence, U.S. Food and Drug Administration, Centers for Medicare & Medicaid Services, International Medical Device Regulators Forum and Singapore Agency for Care Effectiveness. Reference lists of key frameworks and reviews were screened for additional relevant sources. The search was last updated in July 2026.

Search concepts covered health technology assessment, medical devices, CE marking, regulatory approval, market access, comparative effectiveness, economic evaluation, cost-effectiveness, budget impact, real-world evidence, registries, post-market surveillance, hospital-based HTA, procurement, life-cycle HTA, disinvestment, learning curves, device-user interaction, high-risk medical devices, digital health technologies, Software as a Medical Device, artificial intelligence, data drift, managed entry and patient involvement. The complete database-specific search strings are provided in Supplementary File S1. Sources were eligible if they addressed at least one of the following: HTA frameworks for medical devices, methodological challenges, economic evaluation, real-world evidence and post-market assessment, hospital-based HTA or procurement, regulatory–HTA interfaces, patient or public involvement, disinvestment, international implementation, or life-cycle assessment of digital, AI-enabled, or other medical devices. Sources were excluded if they dealt exclusively with pharmaceuticals without transferable methodological relevance, addressed non-health technologies, or lacked conceptual, empirical, methodological, or policy-relevant information.

Sources were selected for their relevance to the review questions and were synthesized narratively. Since this was not a systematic review, the original iterative search was not prospectively logged at record level, and a PRISMA-style flow diagram has not been created. The synthesis included 56 sources: 40 peer-reviewed publications and 16 institutional or policy documents. Because the aim was conceptual synthesis rather than effect estimation, no meta-analysis or formal risk-of-bias assessment was conducted. Table 1 maps the main evidence domains. The synthesis addressed why regulatory conformity is insufficient for determining health-system value, how devices differ from pharmaceuticals for HTA purposes, how uncertainty can affect patient safety, which economic and organizational consequences are hidden by acquisition price, how HTA can be linked to procurement, governance and patient involvement, and how life-cycle HTA can manage uncertainty and support reassessment or disinvestment.

## 3. Results

### 3.1. From Regulatory Conformity to Value-Based Adoption

Regulatory conformity is essential for patient protection; however, it should not be treated as equivalent to health-system value. A medical device that meets regulatory standards may still offer limited incremental benefit when comparing it to existing alternatives, may be difficult to implement safely, may require extensive training or infrastructure, or may incur significant costs that outweigh the value it generates. Conversely, a promising device may deserve controlled implementation prior to widespread diffusion, especially where evidence at market entry is incomplete, and regulatory and reimbursement requirements diverge [7,13,14].

This distinction is particularly important for medical devices, because market access can precede robust evidence on comparative effectiveness, cost-effectiveness and performance in routine care. For example, a diagnostic device may demonstrate analytic validity and/or technical accuracy, yet its value would depend upon whether the test results change clinical practice decisions, improve patient outcomes, reduce unnecessary procedures, or shorten the time to appropriate treatment. Similarly, an implantable device may exhibit satisfactory performance in the short term while its long-term durability, complication rates, revision procedures and patient-related outcomes remain uncertain [13,14,15].

HTA reframes the decision problem. The key question is no longer simply whether the device works according to its intended purpose, but whether it presents a meaningful advantage compared with current standard of care, for the right patient population, at an acceptable cost and within feasible organizational conditions. This comparator-based approach is central to the value-based adoption of medical devices. Additionally, such an approach prevents procurement decisions from being solely dominated by acquisition price, marketing claims, or technical novelty.

The emerging EU HTA framework may strengthen the assessment of selected high-risk devices, but joint clinical assessment alone cannot replace local appraisal. Thus, clinical evidence must still be interpreted relative to local comparators, baseline outcomes, workforce capacity, infrastructure, data systems, reimbursement rules, financial constraints and patient needs. Consequently, the HTA for medical devices should not be viewed as a single report produced at the time of market entry. It should be a decision-support process that begins in early development and follows the device throughout all stages of implementation including follow-up assessments for the purposes of determining continued viability and potential scale-up or withdrawal.

### 3.2. Why Medical Devices Require Dedicated HTA Methods

Medical devices differ from pharmaceuticals in ways that limit the direct transfer of pharmaceutical HTA methods. First, the effectiveness of many devices depends on the interaction between the device and the user. Surgical instruments, imaging equipment, robotic platforms, implantable devices, diagnostic systems and monitoring technologies may all be affected by a variety of factors including operator expertise, team coordination, patient selection and the level of institutional experience. Learning curves and center effects can influence both clinical effectiveness of these products as well as their cost-effectiveness [4,5,6,16,17,18,19,20,21].

Second, medical devices are often embedded in procedures or care pathways rather than functioning as isolated interventions. A device may not be the intervention by itself; rather, it may be one component of a more complex intervention involving protocols, physicians, infrastructure, downstream decisions and follow-up care. For instance, the value of a diagnostic platform depends not only on its technical performance but also on whether positive or negative results lead to better decision making. Likewise, the value of a surgical device depends on the operative technique, perioperative care, rehabilitation, complication management and long-term follow-up [20,21].

Third, medical devices frequently evolve through incremental modification. Hardware, software, materials, accessories, interfaces and algorithms may change over short timeframes. Clinical studies may therefore evaluate a version that has already been modified before study completion. HTA processes must determine when the evidence for one version remains applicable to a subsequent version and when reassessment is required, taking into account the device classification, setting, maintenance and user issues [4,5,6,22]. For SaMD and AI-enabled devices, the assessed object should be version-specific and should include the model, training data, data pipeline, decision thresholds, user interface and workflow. Changes that could affect clinical or subgroup performance should be treated as potential reassessment triggers even when the intended use remains formally unchanged.

Lastly, randomized controlled trials may not be feasible for certain types of medical devices. Blinding may be impractical, sham procedures for evaluation purposes may be unethical, outcomes may be dependent upon the operator experience, and rapid product evolution may make long trials irrelevant. Consequently, medical device HTA typically relies heavily on pragmatic trials, registries, observational studies, post-market evidence and real-world data. Such reliance on non-traditional forms of evidence necessitates methodological flexibility; however, such flexibility must include the explicit assessment of potential bias, confounding, missing data and generalizability [5,6,15,22].

### 3.3. Patient Safety and Clinical Uncertainty

Evidence gaps in medical device assessment can have direct implications for patient safety. A device may perform satisfactorily under controlled conditions but produce different outcomes in routine clinical settings because of user variability, inadequate education and training, inappropriate patient selection, insufficient maintenance, interoperability problems, software updates, or unanticipated workflow effects. For high-risk and implantable devices, harms may emerge only after longer follow-up or broader use in diverse patient populations.

Therefore, the relationship between device performance and patient safety is not simply based on technical considerations; rather, it is also dependent upon organizational factors. Specifically, safe use depends on who uses the device, in which patients, under which protocols, with what training prior to initial use and with what monitoring during ongoing use. A medical device introduced to clinical practice without adequate credentialing regarding the qualifications of personnel using the device, maintenance over time, outcome tracking and feedback mechanisms to identify emerging trends/complications related to the device’s use could potentially generate avoidable risks even if the device itself meets all applicable regulatory requirements. Conversely, structured implementation processes can minimize such risks by ensuring appropriate patient selection for device usage, standardized procedures for utilizing each individual device, definition of clearly assigned responsibilities among healthcare professionals involved in care delivery and the early detection and reporting of complications resulting from device use.

Post-market surveillance and post-market clinical follow-up are essential sources of evidence for understanding how well medical devices perform in actual clinical practice, but they should not be viewed solely as regulatory obligations and remain disconnected from HTA. The Medical Device Coordination Group (MDCG) describes post-market clinical follow-up as a continuous process that updates clinical evaluation and forms part of post-market surveillance planning [23]. For HTA, these data can support the reassessment of the clinical effectiveness, safety, patient-related outcomes, resource use, long-term value created by a medical device and organizational consequences. This logic aligns with total-product life-cycle approaches to medical device regulation and evidence generation, which emphasize continuous learning rather than one-time pre-market assessment [24]. However, post-market data provide valuable insights only if they are collected systematically and used to inform decisions.

Registries, electronic health records, claims data, unique device identifiers, clinical audits and patient-related outcome measures can serve to fill many existing evidence gaps. Device registries are particularly relevant for implantable technologies, where long-term safety, revisions and durability may only become visible after widespread use [25]. However, real-world evidence is not automatically reliable. Data may be incomplete, inconsistently coded, confounded, or disconnected from meaningful outcomes. A recent systematic review focused on real-world evidence for the post-market assessment of medical devices found substantial use of administrative databases, while organizational evidence and patient perspectives were comparatively underreported [26]. Initiatives linking registries, electronic health records and claims data show the potential of real-world evidence to provide meaningful assessments. Nevertheless, these endeavors necessitate clear governance and analytic standards [27]. These findings further reinforce the importance of establishing clear evidence requirements before the adoption of new technologies rather than after uncertainty has become embedded in practice.

Implementing continuous evidence generation is difficult. Device identifiers and software-version information are not consistently recorded in electronic health records, claims and registries, interfaces between systems are fragmented, and data ownership, access and cybersecurity requirements can delay linkage. Registries require long-term funding, specialist data management, clinical engagement, and procedures for completeness and validation. Analytically, treatment selection, center volume, learning curves, time-varying use, missing outcomes and unmeasured confounding can distort real-world estimates. A pragmatic minimum infrastructure includes unique device and version fields, a core data set agreed before adoption, interoperable linkage to outcomes and resource use, a prespecified analysis plan, active comparators, sensitivity analyses and independent governance. The cost of maintaining this infrastructure should be treated as part of the implementation budget or procurement/coverage agreement rather than as an unfunded post-market obligation [25,26,27]. These measures improve interpretability but do not eliminate residual confounding.

### 3.4. Economic and Organizational Consequences

The economic consequences of medical devices extend beyond the acquisition cost. Medical devices may incur additional costs associated with consumables, disposables, software licenses, maintenance contracts, calibration, cybersecurity safeguards, staff training requirements, facility modifications, integration with electronic health records and eventual replacement. In addition, a medical device may also generate downstream costs by increasing the diagnostic yield, procedure volume, frequency of follow-up interventions, or incidental findings. Conversely, a device with a high purchase price may provide good value if it reduces complication and/or admission rates, length of hospital stay, operating time, or unnecessary procedures [18,22,28,29].

These cost structures make economic evaluation more complex than a simple comparison of purchase prices. The same device may have different cost-effectiveness depending on the procedure volume, staff expertise, how efficiently the medical facility operates and the maintenance capacity. For example, a robotic surgical platform may be considered economically unattractive in a low-volume setting but a viable option in a high-volume center with appropriate case mix and operating room efficiency. A remote monitoring device may positively affect outcomes when linked to timely clinical response but may create alert fatigue due to excessive alerts being generated by the device and/or added workload burden if implemented without adequate staffing. Device-specific economic evaluation should therefore incorporate the learning curve effects, maintenance and consumable costs, replacement cycles and organizational consequences [22,29,30].

Organizational consequences are equally important. Medical devices can alter workflow patterns, professional roles, referral pathways, infection-control practices, data governance policies and clinical accountability. They may necessitate new training programs for employees, service contracts with vendors, biomedical engineering support, and/or cybersecurity arrangements. They may also create vendor lock-in through proprietary consumables, software systems, upgrade paths, or maintenance contracts.

Therefore, hospital-based HTA is particularly relevant. Many decisions regarding medical device adoptions occur at a hospital or departmental level, where national HTA may be unavailable, too slow, or does not adequately account for local considerations. Hospital-based HTA can translate general evidence into local decisions by considering the patient volume, current alternative methods, the existing infrastructure, staff capacity, total cost of ownership and risks associated with implementation. The AdHopHTA project emphasized the role of hospital-based HTA in managerial decisions on technology adoption in hospitals [31]. Similarly, NICE medical technology guidance demonstrates how structured assessment frameworks can support hospital-level adoption decisions by linking clinical evidence, cost implications, implementation feasibility and recommendations related to whether or not device-related technologies should be adopted within a given hospital/healthcare delivery system [30,32,33].

### 3.5. Connecting HTA with Procurement and Governance

HTA has limited impact unless it is directly connected to decision-making. For medical devices, this means linking HTA assessment with decisions regarding procurement, reimbursement, clinical governance (i.e., the framework through which healthcare organizations ensure accountability for maintaining and improving the quality and safety of clinical care), quality improvement and disinvestment. Since device adoption is typically decentralized, governance mechanisms must operate at national, regional and hospital levels. The need to align regulatory, reimbursement and assessment processes has been emphasized in previous work on the integrated assessment of medical devices [34], while the disinvestment literature highlights the challenges associated with withdrawing technologies already embedded in practice [35].

At a national level, HTA agencies can prioritize high-risk, high-cost, high-volume, or rapidly diffusing devices. They can also provide methodological guidance on comparators, outcomes, learning curves, real-world evidence, total cost of ownership and uncertainty management. At a payer level, reimbursement can be linked to evidence generation, registry participation, appropriate-use criteria and periodic reassessment. At a hospital level, procurement committees can incorporate HTA principles into purchasing decisions so that adoption is based not only on the acquisition cost and technical specifications but also on the comparative value, implementation capacity, patient safety considerations and life-cycle costs. Medical technology guidance reviews, including those found in NICE medical technologies guidance series, highlight the wide range of evidence domains that can be considered for medical devices [30,32,33].

Multidisciplinary governance is essential. Committees assessing devices should include clinical and patient-safety experts, health economists, biomedical engineers, procurement specialists, nurses, data scientists and patient or public representatives where appropriate. They should evaluate not only whether a device works but also whether the organization is ready to use it safely and efficiently, whether the evidence-generation plan is feasible, and who has the authority to modify or stop use. Conflicts of interest require explicit management, particularly when evidence is vendor-provided or when clinicians involved in the decision have financial or professional relationships with a manufacturer.

Patient and public involvement should be embedded across the life cycle rather than limited to a single committee seat. Patients can help prioritize topics, identify meaningful outcomes and burdens, define acceptable uncertainty, assess usability and equity, shape consent and data-governance arrangements, interpret evidence and communicate conditional or disinvestment decisions. Practical safeguards include accessible evidence summaries, support and reimbursement for contributors, transparent conflict-of-interest procedures and feedback showing how input affected the decision. Patient-reported outcomes and experience measures should be specified before adoption when benefits or burdens may not be captured adequately by clinical endpoints alone.

Procurement contracts can be designed to support HTA [36]. Contracts may include data-sharing requirements, service-level agreements, training obligations, maintenance standards, upgrade transparency, cybersecurity requirements, performance indicators and outcome-based conditions. Managed entry arrangements or risk-sharing agreements may be appropriate when evidence is promising but uncertain, provided they are transparent and do not shift excessive risk to patients or public payers.

Disinvestment is an essential but often neglected component of medical device HTA. Devices may remain in use because of sunk costs, clinician familiarity, contractual obligations, lack of alternatives, or the absence of a decision owner. Operational planning should therefore begin at adoption: conditional decisions should specify the exit criteria, reassessment dates, the authority responsible for acting and a transition plan. Disinvestment should be understood not as indiscriminate rationing, but as releasing resources from technologies that no longer provide acceptable benefit, safety, or value.

When reassessment identifies low value, the response should be proportionate to the urgency and uncertainty. Health systems can update clinical guidelines and appropriate-use criteria, pause new starts, restrict use to defined subgroups or accredited centers, renegotiate or withdraw reimbursement, suspend procurement, decline contract renewal and actively decommission hardware or software. Decommissioning protocols should address patient notification, clinical alternatives, data migration and retention, cybersecurity, maintenance obligations, staff retraining, vendor exit terms and monitoring for unintended consequences. An immediate stop may be required for a credible safety signal, whereas staged restriction may be preferable when the evidence is uncertain, and patients need continuity of care.

### 3.6. A Life-Cycle HTA Approach for Medical Devices

**Figure 1:**
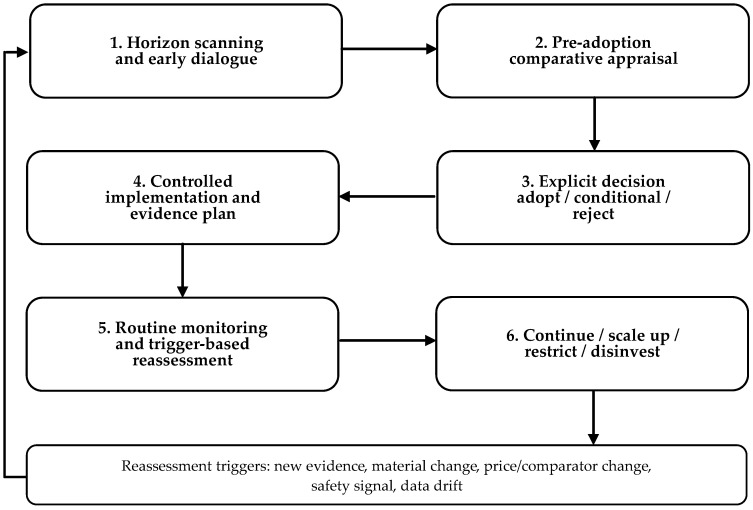
Operational life-cycle HTA pathway for medical devices.

A central problem in medical device adoption is that uncertainty typically persists after regulatory approval and market entry. This supports a transition from one-time assessment to a life-cycle HTA model in which evidence, performance, costs and value are expected to change over time [24,27,37,38,39]. We operationalize this model as six linked phases: (1) horizon scanning and early dialogue, (2) pre-adoption comparative appraisal, (3) an explicit decision to adopt, conditionally adopt, restrict or reject, (4) controlled implementation with training, governance, contracting and an evidence plan, (5) routine real-world monitoring, and (6) scheduled or trigger-based reassessment leading to continuation, scale-up, restriction, renegotiation or disinvestment. Figure 1 provides an overview of the proposed pathway.

Implementation requires a named decision owner, prioritization criteria, predefined comparators and outcomes, an evidence-development plan, data and contractual infrastructure, a reassessment date and authority to act on the findings. National HTA agencies can provide methods and transferable comparative evidence, payers can attach evidence conditions to reimbursement, hospitals can appraise the local readiness and total cost of ownership, procurement teams can embed data, training, service, upgrade and exit requirements in contracts, and registries or data units can generate the agreed upon evidence. Clinicians and patients should define the outcomes, usability concerns and acceptable uncertainty. This distribution of roles converts life-cycle HTA from a sequence of reports into an accountable governance process.

The life-cycle approach also requires methodological flexibility. Randomized trials remain high-quality evidence when feasible, but HTA may also use pragmatic trials, registry-based studies, interrupted time-series analyses, target-trial emulation and linked routine data [40]. These approaches can strengthen causal inference while reflecting real-world practice. Methodological flexibility must not come at the expense of rigor: observational evidence requires the explicit assessment of selection bias, confounding, missing data, measurement error, time-varying exposure and generalizability.

Table 2 operationalizes the proposed framework by specifying the responsibilities, evidence inputs, reassessment triggers, and decision options at each phase.

The same structure can be tailored to different device archetypes. For a high-risk implant, conditional adoption may require unique-device-identifier-linked registry participation and predefined revision-rate or safety thresholds. For a robotic surgical platform, assessment may begin with limited-center use, case-volume and learning-curve targets, total-cost-of-ownership monitoring and staged scale-up. For AI-enabled SaMD, the assessed object includes the model version, data pipeline, decision threshold, interface, workflow and intended population; drift, retraining, or material software changes can trigger reassessment. The framework therefore standardizes governance and decisions without assuming that one evidence package fits all devices.

## 4. Discussion

CE marking and related regulatory mechanisms constitute a necessary but insufficient basis for the adoption of medical devices. Regulatory assessment determines whether a device may be marketed for its intended use, whereas HTA examines whether, for whom, at what cost and under which conditions it should be used in a specific health-system context. This distinction is consistent with comparative analyses of medical device regulation and with the evidence that high-risk devices may enter practice with incomplete or insufficiently transparent comparative data [13,14,36,41,42]. HTA therefore has a complementary role: it translates regulatory evidence into decisions regarding patient benefit, routine-care safety, affordability, implementation requirements and opportunity costs. EU joint clinical assessments may improve the consistency in evidence review; however, local appraisal remains necessary, because capacity, procurement choices and care pathways differ across settings [9,10,11,12,30,31,32,33].

Many of the underlying methodological problems are established in the medical device HTA literature. The added value of the present synthesis is the translation of those problems into a single operational pathway that assigns responsibilities, specifies evidence and implementation inputs, defines scheduled and event-driven reassessment triggers and links findings to procurement, reimbursement, scale-up, restriction and disinvestment. In this sense, the proposed framework complements rather than replaces existing regulatory, HTA, hospital-based HTA and total-product life-cycle models.

The findings of this review further support the need for device-specific HTA methods instead of directly adopting assessment models developed for pharmaceuticals. Medical device performance is often affected by operator skill, learning curves, team experience, infrastructure, maintenance, interoperability and the broader clinical pathway in which the technology is embedded [4,5,6,7,15,16,17,18,22]. Recent recommendations for evaluating high-risk devices similarly emphasize more rigorous clinical investigation, transparent evidence generation, long-term follow-up and appropriate use of registries or pragmatic study designs when randomized trials are difficult or not feasible [43]. Therefore, the economic evaluation of medical devices should extend beyond the acquisition price and consider all components of the total cost of ownership, including the consumables, software licensing fees, training, service contracts, upgrades, downtime and potential downstream consequences for clinical practice [28,29]. In many cases, the most relevant comparator is not simply another product but an alternative diagnostic or therapeutic pathway.

A life-cycle HTA approach offers a practical way to manage these uncertainties. Instead of treating incomplete evidence as a reason either for unrestricted diffusion or for rejection, life-cycle frameworks recommend reassessment of the same device as evidence, prices, practice patterns and alternatives evolve [37,38,39]. This is particularly important for medical devices, since modifications, software updates, expanded indications and accumulating user experience can change the balance of benefit, harm and cost after adoption. Conditional adoption may be appropriate when the expected benefit is plausible, but only if it is linked to predefined outcomes, data collection, a predetermined reassessment date and clear consequences regarding continuation, restriction or withdrawal. Real-world evidence is central to this process, provided that the data sources are fit for the purpose, device identifiers are reliable, outcomes are clinically meaningful and analyses account for bias, confounding, missing data and changes in practice over time [25,44,45].

Therefore, patient safety should be viewed as both a clinical and organizational outcome. Device-related harm may arise not only from malfunctions of the device itself but also from poor patient selection, inadequate training, workflow disruption, maintenance problems, interoperability failures, cybersecurity threats or over-reliance upon automated outputs. Digital and AI-enabled devices intensify these concerns even further, as their performance may vary across populations and settings and may change after software modification or data drift [46,47,48,49,50]. HTA should evaluate whether the organization can operate a device safely and whether patient-related outcomes, burdens, usability issues and equity concerns have been considered. This requires coordination between national HTA agencies, payers, hospitals, procurement teams, clinicians, biomedical engineers, data specialists and patient-safety functions [30,31,32,33].

For AI-enabled SaMD, life-cycle HTA should distinguish a locked model from an adaptive or repeatedly updated system and should treat the model version, training data, data pipeline, decision threshold, interface and intended population as assessment attributes. Monitoring should cover calibration, discrimination, false-positive and false-negative consequences, subgroup performance, workflow effects, clinician overrides and patient outcomes, rather than technical accuracy alone. Changes in population, disease prevalence, scanners, coding, or clinical practice can produce data or concept drift; material performance deviation, retraining, a feature or pipeline change, indication expansion, or a change outside an agreed plan should trigger reassessment. FDA guidance on predetermined change control plans and total-product life-cycle management provides a regulatory basis for bounded risk-based changes with validation, transparency, monitoring, and rollback [51]. HTA adds the question of whether each version continues to deliver comparative clinical and economic value in the local system. Automated compliance checks, machine-readable documentation, and Machine Learning Operations (MLOps)-based monitoring can support auditability, but they do not substitute for the independent evaluation of patient benefit, opportunity cost and equity [52].

Patient and public involvement is also substantive rather than procedural. Patients may identify outcomes, burdens, usability problems, access barriers and distributional effects that are missed by technical or administrative data. Their contribution is relevant to topic prioritization, evidence plans, acceptable uncertainty, conditional adoption, interpretation of real-world findings and communication of restriction or withdrawal decisions. Meaningful participation requires accessible materials, adequate time and support, transparent handling of conflicts and feedback on how the input influenced deliberation [53].

Two examples illustrate why assessment must remain active after market entry. First, stemmed metal-on-metal hip replacements diffused before mature long-term comparative evidence was available. An analysis of 402,051 primary hip replacements in the National Joint Registry of England and Wales found higher revision rates for metal-on-metal devices than for alternative bearings, with the risk increasing with a larger head size [54]. The lesson is not that registries replace pre-market evidence, but that device-linked registries, explicit safety thresholds and timely authority to restrict use are essential. Second, the U.S. Centers for Medicare & Medicaid Services (CMS) coverage policy for transcatheter aortic valve replacement links coverage to a multidisciplinary heart team, institutional and volume requirements and participation in a prospective audited registry that tracks mortality, stroke, repeat procedures, pacemaker implantation, quality of life and long-term durability [55]. This is an operational example of managed entry and evidence generation; it should not be interpreted as proof that every coverage-with-evidence-development arrangement succeeds.

Other jurisdictions illustrate different institutional levers. In the United Kingdom, NICE combines national medical-technology guidance with evidence standards for digital technologies; in the United States, FDA total-product life-cycle and change-control tools address regulatory evolution, while CMS can link coverage to evidence development; Canadian work has explicitly developed life-cycle HTA frameworks; and Singapore’s Agency for Care Effectiveness combines HTA guidance, value-based pricing, tracking of utilization and outcomes, implementation strategies, and patient-facing education [24,30,37,39,46,51,55,56]. These examples suggest convergence around staged evidence generation and reassessment, but responsibility is allocated differently across regulators, HTA bodies, payers and provider organizations.

This review has several limitations. It is a narrative synthesis rather than a systematic review and does not quantify the effectiveness of specific HTA models. The jurisdictional comparison is illustrative rather than comprehensive. The proposed framework is conceptual and has not yet been prospectively evaluated against patient-safety, expenditure, diffusion, or disinvestment outcomes. Device heterogeneity also limits the generalizability of a single framework, and the implementation depends on the data infrastructure, governance capacity, sustainable funding and willingness to act upon reassessment findings. Future research should evaluate which models produce timely evidence and decisions, particularly for high-risk, implantable, digital and AI-enabled technologies [38,39,44,49,50].

## 5. Conclusions

Medical devices are essential to modern healthcare, but responsible adoption requires more than regulatory conformity or CE marking. Their value depends on their comparative clinical benefit, patient safety, user expertise, organizational readiness, infrastructure, workflow integration, maintenance, interoperability, patient experience, total cost of ownership and real-world performance. These dimensions are not captured adequately by one-time regulatory approval or procurement decisions focused mainly on technical specifications and acquisition price.

This review argues that medical devices require dedicated operational life-cycle HTA. The process should begin with horizon scanning and early dialogue, define the relevant comparator and evidence plan to support an explicit decision to adopt, conditionally adopt, restrict, or reject, and link implementation to training, procurement conditions, patient involvement and fit-for-purpose data collection. Scheduled and trigger-based reassessment should then inform continuation, scale-up, renegotiation, restriction, or disinvestment. This is particularly important for high-risk, implantable, expensive, digital and AI-enabled devices, for which uncertainty may persist or evolve after market entry.

Life-cycle HTA should not be viewed as a barrier to innovation. It can create a transparent and accountable pathway for responsible innovation by distinguishing technologies that improve patient outcomes and health-system value from those that add cost, complexity, or risk. The central shift is from technology entry to technology stewardship: CE marking remains essential, but the adoption should be explicit, conditional when appropriate, evidence-generating and revisable. Patient benefit, affordability, equity and accountability should determine whether a device is scaled up, restricted, updated, or withdrawn.

## Figures and Tables

**Table 1:** Thematic map of the sources included in the narrative synthesis.

Evidence Domain	Principal Contribution to the Synthesis	No. of Documents
HTA definitions and life-cycle frameworks	Multidimensional value, timing of assessment, reassessment and implementation of life-cycle HTA	6
Device-specific methods, economics, and surgical innovation	User dependence, learning curves, pathway effects, comparator choice, trial design and total cost of ownership	15
Regulation, market access, and high-risk evidence	Distinction between conformity and value, evidence gaps at market entry, transparency and high-risk device investigation	11
RWE, registries, causal methods and case examples	Post-market evidence, registries, causal inference, safety signals and managed evidence generation	10
Hospital HTA, procurement and disinvestment	Local appraisal, procurement linkage, governance and withdrawal of low-value technologies	5
Digital and AI-enabled devices	Versioned evidence, data drift, continuous monitoring and controlled algorithm modification	7
Patient/public involvement and international implementation	Patient-relevant outcomes, fair deliberation, adoption strategies, outcome tracking and public-facing resources	2

AI: artificial intelligence; HTA: health technology assessment; RWE: real-world evidence.

**Table 2:** Operational life-cycle HTA framework for medical devices. (A) Governance and evidence inputs. (B) Reassessment triggers and decision options.

A. Governance and evidence inputs		
Phase	Lead stakeholders	Core evidence and implementation inputs
1. Horizon scanning and early dialogue	Developers; regulators; HTA agencies; payers; clinicians; patients/public	Unmet need; intended use; target population; comparator; early performance; expected pathway and costs; evidence gaps
2. Pre-adoption comparative appraisal	HTA agency; payer; hospital HTA; procurement; clinical and patient representatives	Regulatory dossier; comparative benefits/harms; economic model; budget impact; equity; patient outcomes; organizational readiness; total cost of ownership
3. Controlled implementation	Hospital leadership; clinical governance; procurement; biomedical engineering; vendor; data team	Credentialing; training; standard protocols; maintenance; interoperability; cybersecurity; contract terms; baseline outcomes; registry/data plan
4. Routine monitoring and evidence generation	Registry/data team; clinicians; patients; safety and quality functions; payer/HTA	UDI and version-linked EHR/claims/registry data; adverse events; PROMs/PREMs; utilization; resource use; maintenance; workflow; subgroup and center performance
5. Formal reassessment	HTA agency; payer; hospital committee; procurement; guideline body; patient/public representatives	Updated comparative evidence; RWE; price and total cost; new alternatives; implementation performance; equity and patient experience
6. Restriction, disinvestment, or transition	Payer; guideline body; hospital leadership; procurement; clinical teams; patients/public	Benefit–risk and value thresholds; alternative pathways; contractual and operational exit plan; transition and communication requirements
B. Reassessment triggers and decision options		
Phase	Scheduled or event-driven triggers	Possible decisions
1. Horizon scanning and early dialogue	High clinical risk; high budget impact; rapid diffusion; major unmet need; disruptive organizational requirements	Prioritize; offer early advice; define evidence plan; defer
2. Pre-adoption comparative appraisal	Market entry; reimbursement request; procurement request; new indication or major upgrade	Adopt; conditional adoption; restrict to subgroup/centers; reject
3. Controlled implementation	First use; new center; new operator group; contract milestone; failure to meet readiness requirements	Pilot; limited-center use; modify implementation; suspend start
4. Routine monitoring and evidence generation	Planned interval; safety signal; performance deviation; learning plateau; utilization or cost variance; data drift; material software/device change	Continue; modify use; require more evidence; temporary restriction
5. Formal reassessment	Predetermined date; new comparator; price change; indication expansion; material modification; persistent uncertainty; contract renewal	Scale up; continue; renegotiate; restrict; initiate disinvestment
6. Restriction, disinvestment, or transition	Unfavorable benefit-risk/value; credible harm; non-compliance with evidence conditions; obsolescence; superior alternative	Stop new starts; withdraw or narrow reimbursement; decommission; replace; monitor transition

EHR: electronic health records, HTA: health technology assessment, PROMs/PREMs: patient-reported outcome measures/patient-reported experience measures, RWE: real-world evidence, UDI: unique device identification.

## Data Availability

No new data were created or analyzed in this study. Data sharing is not applicable to this article.

## Supplementary Materials

The following supporting information can be downloaded at https://www.mdpi.com/article/10.3390/healthcare14142179/s1, Supplementary File S1, Complete database-specific search strategies for PubMed and Scopus.
