# Supplementary material for: Beyond CE Marking: The Need for Life-Cycle Health Technology Assessment of Medical Devices for Patient Safety and Health-System Value
Source: Healthcare (Basel). 2026 Jul 19;14(14):2179. doi: 10.3390/healthcare14142179 (PMC13409966; doi:10.3390/healthcare14142179)
Supplement: Supplementary file 1 [file healthcare-14-02179-s001.zip › healthcare-4356506-supplementary.pdf]

Supplementary File S1

Search Strategy

PubMed: (("health technology assessment"[Title/Abstract] OR HTA[Title/Abstract]) AND ("medical device"[Title/Abstract] OR "medical devices"[Title/Abstract] OR "medical technology"[Title/Abstract] OR "medical technologies"[Title/Abstract] OR "software as a medical device"[Title/Abstract] OR SaMD[Title/Abstract] OR "digital health technology"[Title/Abstract] OR "digital health technologies"[Title/Abstract]) AND ("CE marking"[Title/Abstract] OR regulat*[Title/Abstract] OR "market access"[Title/Abstract] OR adopt*[Title/Abstract] OR procure*[Title/Abstract] OR reimbursement[Title/Abstract] OR "economic evaluation"[Title/Abstract] OR cost-effectiveness[Title/Abstract] OR "budget impact"[Title/Abstract] OR "real-world evidence"[Title/Abstract] OR registr*[Title/Abstract] OR "post-market"[Title/Abstract] OR postmarket[Title/Abstract] OR "hospital-based HTA"[Title/Abstract] OR "life-cycle"[Title/Abstract] OR lifecycle[Title/Abstract] OR disinvestment[Title/Abstract] OR "learning curve"[Title/Abstract] OR "artificial intelligence"[Title/Abstract]))

Scopus: TITLE-ABS-KEY(("health technology assessment" OR HTA) AND ("medical device*" OR "medical technolog*" OR "software as a medical device" OR SaMD OR "digital health technolog*") AND ("CE marking" OR regulat* OR "market access" OR adopt* OR procure* OR reimbursement OR "economic evaluation" OR cost-effectiveness OR "budget impact" OR "real-world evidence" OR registr* OR "post-market" OR postmarket OR "hospital-based HTA" OR "life cycle" OR lifecycle OR disinvestment OR "learning curve" OR "artificial intelligence"))
